# Tradeoffs Between Richness and Bias of Augmented Data in Long-Tail Recognition

**DOI:** 10.3390/e27020201

**Published:** 2025-02-14

**Authors:** Wei Dai, Yanbiao Ma, Jiayi Chen, Xiaohua Chen, Shuo Li

**Affiliations:** 1 School of Telecommunications Engineering, Xidian University, Xi’an 710071, China; 22012100039@stu.xidian.edu.cn (W.D.); 22012100031@stu.xidian.edu.cn (J.C.); 2 School of Artificial Intelligence, Xidian University, Xi’an 710071, China; lishuo@xidian.edu.cn; 3 Department of Automation, Tsinghua University, Beijing 100190, China; chenxiaohua@mail.tsinghua.edu.cn

**Keywords:** representational learning, long-tail distribution, image recognition, data-centric AI

## Abstract

In long-tail scenarios, models have a very high demand for high-quality data. Information augmentation, as an important class of data-centric methods, has been proposed to improve model performance by expanding the richness and quantity of samples in tail classes. However, the underlying mechanisms behind the effectiveness of information augmentation methods remain underexplored. This has led to reliance on empirical and intricate fine-tuning in the use of information augmentation for long-tail recognition tasks. In this work, we simultaneously consider the richness gain and distribution shift introduced by information augmentation methods and propose effective information gain (EIG) to explore the mechanisms behind the effectiveness of these methods. We find that when the value of the effective information gain appropriately balances the richness gain and distribution shift, the performance of information augmentation methods is fully realized. Comprehensive experiments on long-tail benchmark datasets CIFAR-10-LT, CIFAR-100-LT, and ImageNet-LT demonstrate that using effective information gain to filter augmented data can further enhance model performance without any modifications to the model’s architecture. Therefore, in addition to proposing new model architectures, data-centric approaches also hold significant potential in the field of long-tail recognition.

## 1. Introduction

**Figure 1:**
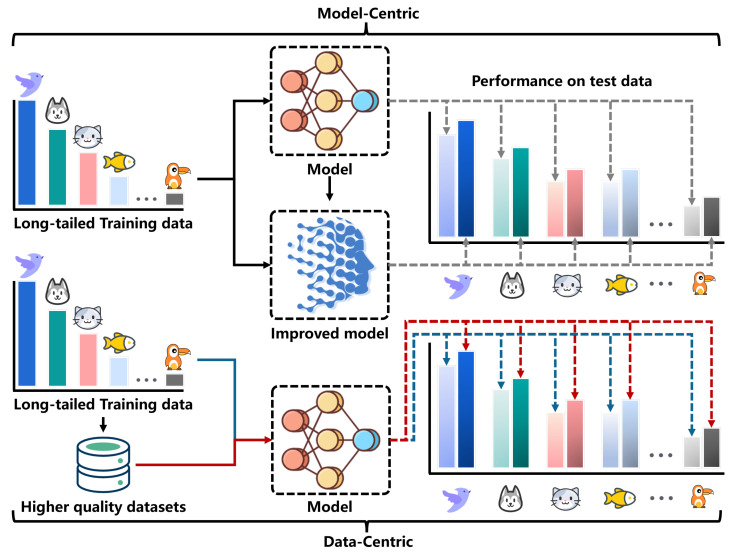
In the traditional development cycle of AI models, researchers primarily focus on improving the model’s architecture or training techniques to enhance the performance of long-tail recognition. The common research paradigm involves assessing performance differences between different models, given training and test data. Data-centric long-tail learning, on the other hand, should keep the model constant and concentrate on improving the quality of the dataset.

Long-tail data distributions are common in real-world applications, where imbalanced data distributions often lead to poor model performance in certain categories [1,2,3,4,5,6,7,8,9]. Long-tail datasets are characterized by a few classes having many samples (the ‘head’), while most classes are underrepresented, with only a few samples (the ‘tail’). This imbalance often leads to poor model performance in the underrepresented classes, which is a major challenge in machine-learning tasks, such as image classification and image recognition [10,11,12]. To overcome this bias, a range of long-tail learning methods have emerged, which can be broadly classified into two categories: model-centric and data-centric approaches. As illustrated in Figure 1, model-centric methods aim to improve the model’s structure to enhance performance in long-tail recognition. Examples of such methods include decoupled training strategies [13,14,15,16] and reweighted loss functions [17,18,19,20,21,22,23,24], among others. In contrast, data-centric information augmentation focuses on improving model performance by enhancing the quality of the training data, particularly for the tail categories, by increasing their sample representation. This category includes data augmentation techniques, like Mixup, Cutout, and CutMix, as well as knowledge-guided tail augmentation methods, such as OFA [25], CMO [26], and FDC [27]. Although previous research has predominantly concentrated on improving model structures for long-tail recognition, the benefits of structural enhancements have diminished with the rapid development of foundational models [28,29]. Consequently, data-centric approaches to long-tail recognition have gained increasing importance [30,31,32]. However, the effectiveness of information augmentation methods often relies on empirical experience rather than quantifiable metrics [33].

Different information augmentation methods perform significantly differently in various circumstances. When the samples of tail categories adequately cover their true distributions (Definition 2), traditional data augmentation methods usually perform well. However, in reality, tail category samples are often limited and fail to sufficiently represent their true distributions, rendering conventional augmentation methods as less effective [25,27]. This necessitates the introduction of additional knowledge to expand the samples of tail categories and facilitate the model’s learning of latent distributions (Definition 3). For example, FDC [27] re-estimates tail distributions using the variance of the head distribution, while CMO and OFA combine background information from head samples with foreground information from tail samples to generate more diverse tail samples.

However, the current use of information augmentation methods heavily relies on empirical knowledge and parameter tuning, lacking quantitative research on how to generate and select appropriate augmented samples. When augmented samples deviate beyond the real distribution, they may conflict with sample labels, thus interfering with the model’s learning process [17,34,35,36]. Conversely, when augmented samples remain within the observed distribution (Definition 1), they fail to provide new information for tail classes. Therefore, the selection of high-quality augmented samples seems to be a tradeoff between richness gain and distribution shift.

In this work, we propose a model-independent metric called effective information gain (EIG), which simultaneously considers the richness gain and distribution shift introduced by augmented data (see Section 2). Traditional measures of information gain, such as mutual information, primarily focus on information content, quantifying the amount of shared information between features. In contrast, we introduce the concept of effective information gain (EIG) to quantify the true effectiveness of augmented data. The underlying goal is to measure the degree of non-overlap between two distributions, offering a more nuanced perspective on data augmentation. Unlike mutual information, which emphasizes information quantity, EIG considers both the richness of features and the model’s ability to adapt to long-tail distributions. This distinction is crucial in the context of long-tail recognition, where the objectives are not only to maximize information content but also to enhance the model’s ability to generalize to underrepresented classes. Through extensive experiments, we observed that the performance of information augmentation methods is maximized when the EIG of the augmented data is within an appropriate range. We further explore the relationship between data imbalance and the optimal EIG and propose an algorithm to select augmented data with a specified EIG level in practical applications. This work provides a systematic tool for the evaluation and application of information augmentation methods in the field of long-tail image recognition. Moreover, by selecting augmented data with appropriate EIG levels generated by various state-of-the-art information augmentation methods, we further enhance the long-tail recognition performance of models in the large-scale ImageNet-LT dataset. Comprehensive experimental results confirm our analysis that information augmentation methods achieve optimal performance when the EIG values of the augmented samples are within a reasonable range (Section 3). This work represents the first successful attempt to apply a data-centric approach in the field of long-tail recognition, aiming to draw the attention of researchers to this promising direction.

### Information Augmentation

When the few samples of the tail class do not represent its true distribution well, the model cannot learn information outside the observed distribution [25,27], no matter how one goes about adjusting the sampling strategy and balancing the losses. Information augmentation facilitates imbalance learning by introducing additional knowledge and can be divided into two types of methods: knowledge transfer and data augmentation.

Head-to-tail knowledge transfer aims to transfer knowledge from the head class to the tail class to improve the performance of the tail class. FTL [37], FDC [27], and LEAP [38] generate enhancement samples by transferring the head class’s variance to the tail class. It is worth noting that FDC provides the experimental foundation for transferring variance for the first time. OFA [25] decomposes the features of each class into the class’s general features and class-specific features. During training, the class-specific features of the tail class are fused with the generic features of the head class to generate new features to augment the tail class. GIST [39] proposes to transfer the geometric information of the feature distribution boundaries of the head class to the tail class. The motivation of CMO [26] is very intuitive; it considers that the images of the head class have a rich background, so the images of the tail class can be directly pasted onto the rich background of the head class to increase the richness of the tail class. CUDA [40] gives the appropriate enhancement intensity for each class, through empirical studies, which can be combined with other augmentation methods.

Data augmentation in long-tail recognition improves the performance of tail classes by improving conventional data augmentation methods. MiSLAS [41] and Remix [42] suggest employing Mixup to enhance feature learning. Recent research [43] has found that Mixuphas good accuracy when combined with resampling. FASA [44] proposes generating features based on Gaussian priors and evaluating weak classes in balanced datasets to adjust the sampling rate. MetaSAug [45] generates enhanced features for tail classes with ISDA. Recent studies show that noise-based data augmentation can also play an important role in long-tail recognition. Similar to adversarial attacks, M2m [46], AdvProp [47], and FedAFA [48] propose to transform some of the head class samples to tail class samples by perturbation-based optimization to achieve tail class augmentation. Unlike adversarial noise, OpeN [49] attempts to enhance tail classes directly using pure noise images, which outperform relative to the adversarial noise-based approach.

Unlike the methods mentioned above, OUR [50] identified and defined the long-tail phenomenon of model robustness, expanding the research scope of long-tail learning. Furthermore, OUR introduced orthogonal uncertainty representations of manifolds for augmenting tail classes, significantly enhancing the model’s robustness in tail classes.

## 2. Data-Centric Performance Metrics

We aim to construct a data-dependent metric to guide and enhance the performance of existing long-tail information augmentation methods. This section is organized as follows: First, we discuss the potential mechanisms related to the performance of information augmentation methods from the perspective of data manifolds. Next, we introduce the concept of effective information gain (EIG) to quantify the richness gain and distribution shift induced by information augmentation methods. It is important to note that our metric is directly computed from the data distribution and does not rely on the model.

### 2.1. Inspiration from Marginal Effects

First, we define the concepts of observed distribution, true distribution, and underlying distribution.

**Definition** **1** (Observed Distribution)**.** *The distribution formed by the available samples (i.e., the training set).*

**Definition** **2** (True Distribution)**.** *The distribution formed by samples when there are enough samples of a class to fully encompass all the characteristics of that class.*

**Definition** **3** (Underlying Distribution)**.** *The true distribution other than the observed distribution.*

The manifold distribution law [18,51] suggests that samples in the same class of natural data are located near a low-dimensional manifold embedded in a high-dimensional space, while samples in different classes are distributed near different manifolds. DSB [17] measures the diversity of class features by the volume of the manifold and points out that decision boundaries often favor classes with lower volumes, resulting in model bias. However, in long-tail scenarios, the observed distribution of tail classes usually fails to adequately cover their true distribution. To address this issue, information augmentation methods increase the richness of tail class samples, expanding the volume of their observed distribution and, thus, enabling the model to learn more information beyond the training domain. Therefore, we reasonably infer that **the richness gain provided by information augmentation for tail classes may be the underlying mechanism for its effectiveness.**

**Figure 2:**
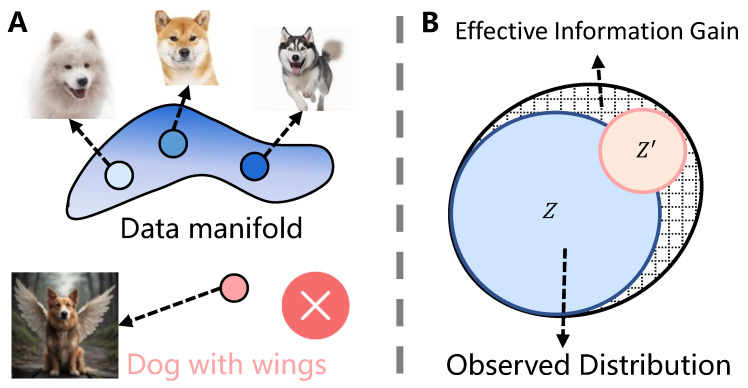
(**A**) The data manifold corresponding to a class has boundaries, and samples outside the manifold boundaries may not exist in the real world, such as dogs with long wings. (**B**) Grid regions represent the diversity gain in features brought about by augmented samples.

The marginal effect [19,50] suggests that the diversity of features for each class is limited; in other words, data manifolds have boundaries, and samples that lie beyond these boundaries do not exist in the real world (as shown in Figure 2A). Information augmentation methods may generate samples that fall outside the range of the true distribution. When the newly generated samples deviate too far from the observed distribution, this can cause the data manifold of the tail classes to overlap with other manifolds, thereby harming the model’s performance [27]. Therefore, we are interested in studying the impact of the deviation degree between augmented samples generated by information augmentation methods and the observed distribution on the model’s performance. **In the following section**, we will focus on proposing a metric that reflects both the richness gain and distribution shift.

### 2.2. Effective Information Gain (EIG)

Data augmentation methods can generate augmented samples both in the image space and in the embedded space. To maintain the generality of the proposed metric, we assume the dimensionality of the samples to be *d* uniformly. Below, we employ the volume of the data manifold to measure the category richness and, subsequently, define the effective information gain (EIG).

Given the class of data $X=\left[x_{1},x_{2},\ldots ,x_{k}\right]\in R^{d\times N}$, where *d* is the dimensionality of the samples, and *N* is the number of samples in that category, we first estimate the covariance matrix $\Sigma =E\left[\frac{1}{N}\sum_{i=1}^{N}x_{i}x_{i}^{T}\right]=\frac{1}{N}XX^{T}\in R^{d\times d}$ of the sample set *X*. Next, the volume of the subspace spanned by the sample set *X* can be succinctly expressed as $\sqrt{\det(\frac{1}{N}XX^{T})}$, which can be obtained through the singular value decomposition of matrix *X*. To address situations where $\frac{1}{N}XX^{T}$ is not full rank and leads to a volume of 0, an identity matrix is added to the covariance matrix, without affecting the monotonicity of the volume. Furthermore, for enhanced numerical stability, the volume of the data manifold corresponding to the logarithmically transformed sample set *X* can be calculated as

$$V\left(X\right)=\frac{1}{2}\log_{2}\det\left(I+\frac{1}{N}XX^{T}\right).$$

The result of the above calculation can be regarded as a measure of the feature richness of the sample set *X*. On this basis, we introduce the definition of the effective information gain (EIG). The effective information gain represents the increase in data richness for a particular category when using an information augmentation method. Additionally, we expect this metric to reflect the degree of deviation between the distribution of the augmented samples and the original samples.

In the image space, the richness of a sample set is fixed. However, in the embedding space, we observe that the magnitude of the feature richness is uncertain when different models extract feature embeddings from the same sample set. This uncertainty could be because of the randomness of the parameter initialization. Given that multiple experiments were conducted in this study to observe the relationship between the effective information gain and the performance of the information augmentation methods, we aimed for the quantitative results of the effective information gain to be unaffected by the magnitude of the feature richness.

Motivated by this, we define the effective information gain in the form of a ratio. Specifically, the EIG can be measured as the increase in the ratio of the feature richness of the augmented dataset (after applying the augmentation), minus the initial feature richness, to the initial feature richness of the samples. For a more intuitive explanation, in Figure 2B, the blue region represents the distribution of the initial samples (Z), the yellow region represents the distribution of the augmented samples, and the grid area represents the increase in the feature richness brought about by applying the information augmentation method. The proposed metric should satisfy the following principles:

- **Principle 1:** When the number of augmented samples is 0, the effective information gain is 0.
- **Principle 2:** Regardless of the number of augmented samples, if all the augmented samples are concentrated at a single point, they do not provide any additional information and, instead, increase the training cost. Therefore, we expect the EIG to be negative in this case, reaching its minimum value (lower bound).

On the basis of the above principles, we formally define the measure of the EIG as follows.

**Definition** **4** (**Effective Information Gain**)**.** *Let A be an information augmentation method, and $X^{\prime }$ be the augmented samples generated from X using method A, with a count of $N^{\prime }$. Let M denote the model trained using both $X^{\prime }$ and X, and $Z^{\prime }$ and Z represent the d-dimensional features obtained from M corresponding to $X^{\prime }$ and X, respectively. Let $F=\left[Z,Z^{\prime }\right]\in R^{d\times (N+N^{\prime })}$, and perform mean normalization on Z, $Z^{\prime }$, and F. We define the effective information gain (EIG) after augmentation using method A as follows:*

$$\begin{array}{cc} \; & EIG\left(A,F,Z\right)=\frac{V(F)-V(Z)}{V(Z)}\\ \; & =\frac{\log_{2}\det\left(I+\frac{1}{N+N^{\prime }}FF^{T}\right)-\log_{2}\det\left(I+\frac{1}{N}ZZ^{T}\right)}{\log_{2}\det\left(I+\frac{1}{N}ZZ^{T}\right)}\\ \; & =\frac{\log_{2}\det\left(I+\frac{1}{N+N^{\prime }}FF^{T}\right)\det{\left(I+\frac{1}{N}ZZ^{T}\right)}^{-1}}{\log_{2}\det\left(I+\frac{1}{N}ZZ^{T}\right)}\\ \; & =\log_{\delta }\frac{\det(I+\frac{1}{N+N^{\prime }}FF^{T})}{\det(I+\frac{1}{N}ZZ^{T})},\\ \delta & =\det(I+\frac{1}{N}ZZ^{T}), \end{array}$$

*where V(F) denotes the volume of F, and V(Z) denotes the volume of Z. In the following section, we verify that the EIG satisfies the two principles one by one:*

(1) EIG(A,F,Z) can be expressed as
  $$\log_{\delta }\frac{\det(I+\frac{1}{N+N^{\prime }}\left(ZZ^{T}+Z^{\prime }Z^{\prime T}\right))}{\det(I+\frac{1}{N}ZZ^{T})}.$$
  When $N^{\prime }=0$, $Z^{\prime }Z^{\prime T}$ is a zero matrix, so $EIG\left(A,F,Z\right)\;=\;log_{\delta }1\;=\;0$, which satisfies Principle 1.
(2) We prove that when all the augmented samples converge to a single point, EIG reaches the lower bound $-\frac{N^{\prime }}{N+N^{\prime }}$, thus fulfilling Principle 2. The proof is outlined below.

**Proof.**  The function logdet(·) is strictly concave and satisfies the following inequality:

$$\log\det\left(\sum_{j=1}^{k}\alpha_{j}S_{j}\right)\geq \sum_{j=1}^{k}\alpha_{j}\log\det\left(S_{j}\right),$$

where $\alpha_{j}>0$, $\sum_{j=1}^{k}\alpha_{j}=1,j=1,\ldots ,k$, and $S_{j}\left(j=1,\ldots ,k\right)$ are symmetric positive-definite matrices. When k=2, we have

$$\log\det\left(\alpha_{1}S_{1}+\alpha_{2}S_{2}\right)\geq \alpha_{1}\log\det\left(S_{1}\right)+\alpha_{2}\log\det\left(S_{2}\right).$$

By setting $\alpha_{1}=\frac{N}{N+N^{\prime }}$, $\alpha_{2}=\frac{N^{\prime }}{N+N^{\prime }}$, $S_{1}=I+\frac{1}{N}ZZ^{T}$, and $S_{2}=I+\frac{1}{N^{\prime }}Z^{\prime }Z^{\prime T}$, we can derive

$$\begin{array}{cc} \log\det(\alpha_{1}S_{1}\;+\;\alpha_{2}S_{2}) & =\log\det(I\;+\;\frac{1}{N+N^{\prime }}\left(ZZ^{T}\;+\;Z^{\prime }Z^{\prime T}\right))\\ \; & =\log\det(I+\frac{1}{N+N^{\prime }}FF^{T}). \end{array}$$

Thus, we have

$$\begin{array}{cc} \; & \log\det\left(I+\frac{1}{N+N^{\prime }}FF^{T}\right)\geq \frac{N}{N+N^{\prime }}\log\det\left(I+\frac{1}{N}ZZ^{T}\right)\\ \; & +\frac{N^{\prime }}{N+N^{\prime }}\log\det\left(I+\frac{1}{N^{\prime }}Z^{\prime }Z^{\prime T}\right)\\ \; & ⟹\frac{1}{2}\log\det\left(I+\frac{1}{N+N^{\prime }}FF^{T}\right)\\ \; & \geq \frac{1}{N+N^{\prime }}\left(\frac{N}{2}\log\det\left(I+\frac{1}{N}ZZ^{T}\right)+\frac{N^{\prime }}{2}\log\det\left(I+\frac{1}{N^{\prime }}Z^{\prime }Z^{\prime T}\right)\right)\\ \; & ⟹V\left(F\right)\geq \frac{1}{N+N^{\prime }}\left(NV\left(Z\right)+N^{\prime }V\left(Z^{\prime }\right)\right). \end{array}$$

Further it can be obtained that

$$\begin{array}{c} \frac{V(F)-V(Z)}{V(Z)}\geq \frac{\left(\frac{N}{N+N^{\prime }}-\frac{N+N^{\prime }}{N+N^{\prime }}\right)V\left(Z\right)+\frac{N^{\prime }}{N+N^{\prime }}V\left(Z^{\prime }\right)}{V(Z)}\\ ⟹\frac{V(F)-V(Z)}{V(Z)}\geq \frac{\frac{-N^{\prime }}{N+N^{\prime }}V\left(Z\right)+\frac{N^{\prime }}{N+N^{\prime }}V\left(Z^{\prime }\right)}{V(Z)}. \end{array}$$

Again, because $I+Z^{\prime }$ is a positive-definite matrix, $V(Z^{\prime })\geq 0$. When the samples in $Z^{\prime }$ are concentrated at one point, $\frac{V(F)-V(Z)}{V(Z)}$ reaches a **lower bound** $\frac{-N^{\prime }}{N+N^{\prime }}$ because $V(Z^{\prime })=0$.    □

It is worth mentioning that [33] assumes that the feature richness after augmentation is obtained by adding the feature richness values of the initial samples and the augmented samples. However, we argue that the feature richness after augmentation should be measured by the volume of the subspace spanned jointly by both the augmented and initial samples, rather than the sum of the volumes of their individually spanned subspaces. This approach helps to avoid the separation of features in the same class by the decision boundary. Additionally, it has the benefit of allowing the effective information gain (EIG) to reflect the degree of the distribution shift between the augmented and original samples.

## 3. Empirical Study and Analysis

In this section, we will explore the relationship between the effective information gain (EIG) and the performances of the data augmentation methods. According to the hypothesis and analysis in Section 2.1, a data augmentation method should maximize its performance when the EIG is within an appropriate range. When the EIG is too low, it indicates that the additional information brought by the data augmentation method is minimal. Conversely, when the EIG is too high, it may result in augmented samples that lack practical significance. Therefore, both excessively high and low EIGs can limit the performances of data augmentation methods.

**Figure 3:**
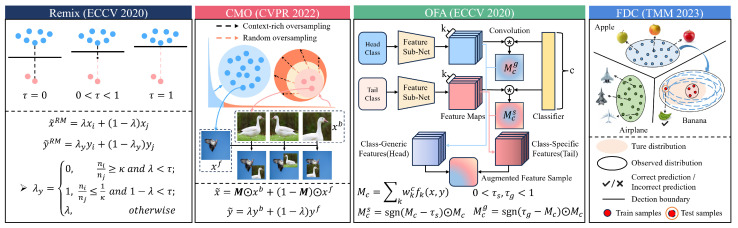
**Remix** improves upon Mixup by modifying the synthesized labels so that when samples from head classes and tail classes are mixed, the resulting label is 100% contributed by the tail class. **CMO** uses CutMix to paste patches from tail class samples onto head class samples, thereby generating augmented samples. **OFA** decomposes sample features into class-specific and class-generic features and combines the tail class’s specific features with the head class’s generic features to generate augmented samples for the tail class. **FDC** observes that similar classes have similar distribution statistics (variances) and, thus, transfers the variance of the most similar head class to the tail class to re-estimate the distribution, sampling augmented samples from the new distribution.

To validate this hypothesis, we conducted experiments on the commonly used long-tail datasets CIFAR-10-LT, CIFAR-100-LT, and ImageNet-LT. Information augmentation can be performed in both the image space and embedding space. In the image space, we selected the representative methods Remix [42] and CMO [26] for investigation. In the embedding space, we chose the advanced methods OFA [25] and FDC [27] for analysis. All four methods generate augmented samples for tail classes to balance the training data. Figure 3 illustrates the core operations of these four data augmentation methods.

### 3.1. Datasets and Evaluation Metrics

CIFAR-10-LT and CIFAR-100-LT [17,19] are long-tail datasets, including five imbalance factors (IF = 10, 20, 50, 100, and 200) generated based on CIFAR-10 and CIFAR-100. The imbalance factor (IF) is defined as the number of the most frequent class training samples divided by the number of the least frequent class training samples. ImageNet-LT [12,18] is a long-tail subset of ILSVRC 2012, with an imbalance factor of 256, which contains 1000 classes totaling 115.8 k images, with a maximum of 1280 images and a minimum of 5 images per class. The balanced 50 k images were used for testing. For a fair comparison, the training and test images of all the datasets are officially split, and the top accuracy in the test set is utilized as a performance metric.

### 3.2. Implementation Details

For CIFAR-10-LT and CIFAR-100-LT, we trained ResNet-32 using the SGD optimizer, with a momentum of 0.9 and a weight decay of 0.0002. In ImageNet-LT, we used ResNeXt-50 as the backbone for all the methods. The models were trained with the SGD optimizer, a batch size of 256, a momentum of 0.9, a weight decay factor of 0.0005, and a learning rate of 0.1 (with linear LR decay).

### 3.3. Utilization of Remix, CMO, OFA, and FDC

Remix and CMO directly augment the tail classes in the image space and then proceed with training. OFA and FDC both employ decoupled training [14], thus the training phase consists of two parts. After the first phase concludes, OFA and FDC augment the tail classes in the embedding space and then feed the augmented samples into the second phase for training. When using Remix, CMO, and OFA, it is necessary to select sample pairs to generate augmented samples. Assuming the number of samples in tail class *t* is $n_{t}$, if the sample count of a category exceeds $3n_{t}$, Remix considers that category as a relative head class compared to tail class *t*. Furthermore, Remix matches a head class sample for each sample from the tail class to create sample pairs. CMO directly samples background samples from the long-tail distribution, while foreground samples are selected from the tail class. OFA matches a sample from the most similar head class for each tail class sample to create sample pairs. FDC controls the quantity of the augmented samples by altering the sampling frequency.

Whether in the image space or the embedding space, our goal is to use the four methods mentioned above to balance the long-tail dataset. Taking CIFAR-10-LT (IF = 100) as an example, the class with the most samples has 5000 samples, while the class with the fewest samples contains only 500 samples. We need to generate 4500 augmented samples for the class with the fewest samples in the tail. Similarly, the sample count for the other tail classes also needs to be increased to 5000. Herein, we introduce the method described in [25] for partitioning head and tail classes. First, the categories are sorted in descending order of sample quantity, with the first *h* categories selected as head classes and the rest as tail classes. The ratio of the number of head class samples to the total number of samples is defined as $h_{r}\in \left(0,1\right)$, and *h* is selected as the minimum value that satisfies $h_{r}>0.9$.

### 3.4. Generating Augmented Data Containing Diverse EIG Values

To investigate the impact of the effective information gain (EIG), brought by augmented data, on the model’s performance, multiple sets of augmented data with varying EIGs need to be generated. Taking Remix as an example, a single application of Remix can generate sufficient augmented samples for tail classes in long-tail datasets. The average effective information gain for tail classes is defined as ${\mathrm{EIG}}_{\mathrm{Tail}}=\frac{1}{\left|\mathrm{Tail}\;\mathrm{class}\right|}\sum_{t\in \mathrm{Tail}\;\mathrm{class}}{\mathrm{EIG}}_{t}$, where ${\mathrm{EIG}}_{t}$ represents the effective information gain brought by the augmentation method to tail class *t*, and |Tailclass| denotes the number of tail classes. In this study, we first used Remix 100 times to generate 100 sets of different augmented data and calculated their corresponding ${\mathrm{EIG}}_{\mathrm{Tail}}$ values. We sorted these 100 ${\mathrm{EIG}}_{\mathrm{Tail}}$ values in descending order and sampled 50 uniformly distributed ${\mathrm{EIG}}_{\mathrm{Tail}}$ values. However, cases with lower or higher ${\mathrm{EIG}}_{\mathrm{Tail}}$ values were rare among these 50 sets.

To obtain augmented datasets with a wide range of effective information gains, we propose the following data selection process (Algorithm 1). First, we generate a high number of augmented samples and cluster these samples into multiple subsets using *k*-means clustering (500 subsets in this study). Assuming each subset contains 10 samples, if a specific tail class requires 450 augmented samples, we need to sequentially select 45 subsets for that tail class. In the subset selection process, to generate augmented data with higher EIGs, we design a gain prioritization strategy. This strategy prioritizes subsets that maximize the EIG at each step of the subset selection. Conversely, to generate augmented data with lower EIGs, we select subsets that minimize the EIG. By dynamically selecting different subsets, we can create augmented datasets with varying EIG values.
**Algorithm 1** Subset selection algorithm for diverse EIG-augmented data**Require:** Target EIG ranges ${\mathrm{EIG}}_{\min}$ and ${\mathrm{EIG}}_{\max}$, subsets $S=\{S_{1},S_{2},\ldots ,S_{n}\}$, subset EIG values $\mathrm{EIG}(S_{i})$, tolerance ϵ, and the number of desired subsets for each range (N)**Ensure:** Augmented datasets $D_{\mathrm{aug}}^{\mathrm{large}}$, $D_{\mathrm{aug}}^{\mathrm{small}}$, and $D_{\mathrm{aug}}^{\mathrm{diverse}}$1:Generate a high number of augmented samples and cluster them into subsets S using k-means2:Initialize $D_{\mathrm{aug}}^{\mathrm{large}}\leftarrow \emptyset$3:Initialize $D_{\mathrm{aug}}^{\mathrm{small}}\leftarrow \emptyset$4:Initialize $D_{\mathrm{aug}}^{\mathrm{diverse}}\leftarrow \emptyset$5:Calculate initial EIG for each subset in S6:Sort subsets by their EIG values in ascending order: $\mathrm{sorted}\_\mathrm{subsets}\leftarrow \mathrm{sort}(S,\mathrm{key}=\mathrm{EIG}\left(S_{i}\right))$7:Select the top subsets for a wide EIG range8:**for** 
i←1 **to** *N* **do**9:   Find subset $S_{k}$ that maximizes $|\mathrm{EIG}\left(D_{\mathrm{aug}}^{\mathrm{large}}\cup S_{k}\right)-{\mathrm{EIG}}_{\max}|$10:   Update $D_{\mathrm{aug}}^{\mathrm{large}}\leftarrow D_{\mathrm{aug}}^{\mathrm{large}}\cup S_{k}$11:   Update EIG values accordingly12:   Remove $S_{k}$ from S13:**end for**14:Select bottom subsets for narrow EIG range15:**for** i←1 **to** *N* **do**16:   Find the subset $S_{k}$ that minimizes $\left|\mathrm{EIG}\left(D_{\mathrm{aug}}^{\mathrm{small}}\cup S_{k}\right)-{\mathrm{EIG}}_{\min}\right|$17:   Update $D_{\mathrm{aug}}^{\mathrm{small}}\leftarrow D_{\mathrm{aug}}^{\mathrm{small}}\cup S_{k}$18:   Update EIG values accordingly19:   Remove $S_{k}$ from S20:**end for**21:Ensure diversity in EIG values22:Calculate EIG ranges of all the subsets in S23:Select subsets to cover diverse EIG values: $D_{\mathrm{aug}}^{\mathrm{diverse}}$24:**return** $D_{\mathrm{aug}}^{\mathrm{large}}$, $D_{\mathrm{aug}}^{\mathrm{small}}$, and $D_{\mathrm{aug}}^{\mathrm{diverse}}$

Following this process, we generated 50 sets of augmented data with high ${\mathrm{EIG}}_{\mathrm{Tail}}$ values and 50 sets with low ${\mathrm{EIG}}_{\mathrm{Tail}}$ values, totaling 100 sets. From these 100 sets, we selected 50 sets with evenly distributed ${\mathrm{EIG}}_{\mathrm{Tail}}$ values for experimentation. Similarly, for each long-tail dataset, we retained 50 sets of augmented data for each of the three augmentation methods—CMO, OFA, and FDC—to ensure comprehensive and comparative experimentation.

### 3.5. Augmented Data Require Appropriate EIGs

We chose to conduct classification experiments using four information augmentation methods on six different datasets: CIFAR-10-LT (IF = 10, 50, and 100) and CIFAR-100-LT (IF = 10, 50, and 100) [19]. Each of the four methods generated 50 sets of effective augmented data for each dataset, resulting in a total of 1200 classification experiments.

**Figure 4:**
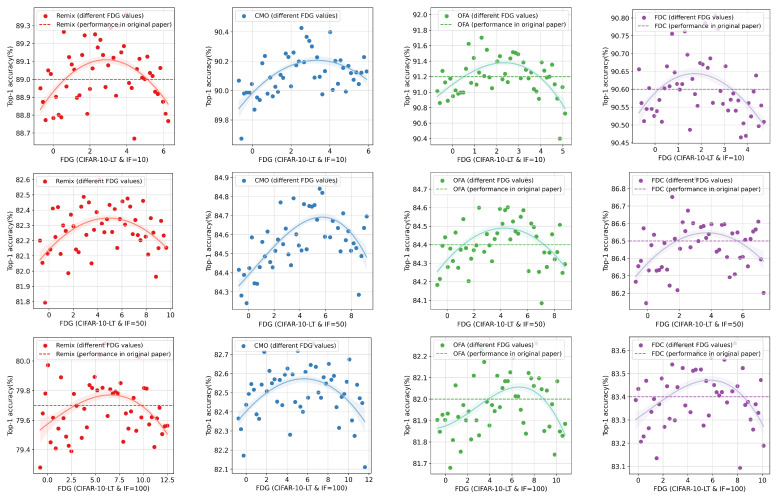
The performances of information augmentation with different FDGs in CIFAR-10-LT.

**Figure 5:**
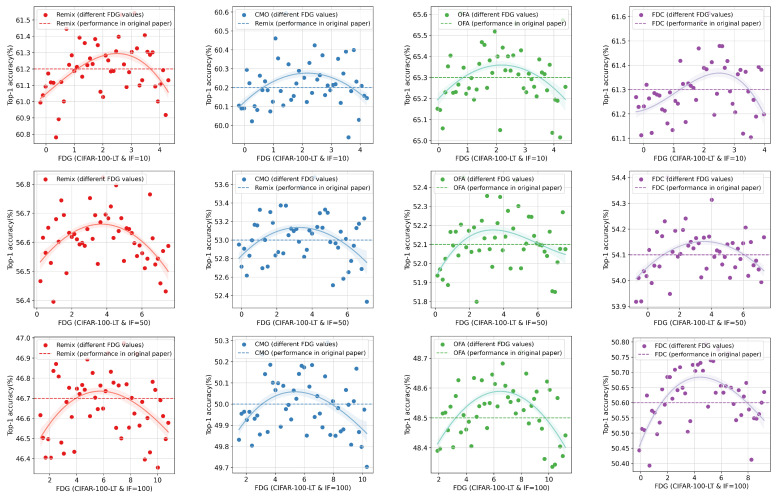
The performances of information augmentation with different FDGs in CIFAR-100-LT.

The experimental results for CIFAR-10-LT are shown in Figure 4. Overall, it can be observed that when the ${\mathrm{EIG}}_{\mathrm{Tail}}$ of the augmented data falls within an appropriate range, the performance of the information augmentation method reaches its optimum, surpassing the results reported using the original method. **This result confirms our hypothesis that effective augmented data require a balance between the richness gain and the distribution shift it introduces**. Moreover, as the imbalance level of the dataset increases, the data augmentation method needs to generate augmented data that provide greater EIGs. The experimental findings for CIFAR-100-LT (see Figure 5) are largely consistent with those for CIFAR-10-LT. It is worth noting that the original paper on CMO did not provide results for CIFAR-10-LT. Therefore, we only conducted classification experiments and did not provide a benchmark for comparison, but this does not hinder the primary conclusions of our experiments. Our results indicate that guiding data augmentation methods to generate more appropriate samples can further improve the performances of long-tail recognition models.

### 3.6. Relationship Between Data Imbalance and the Optimal EIG

Previously, we demonstrated through experiments that a good set of augmented data should fall within an appropriate ${\mathrm{EIG}}_{\mathrm{Tail}}$ range. Next, we attempt to establish a connection between data imbalance levels and ${\mathrm{EIG}}_{\mathrm{Tail}}$, which will allow the EIG to serve as a stronger indicator for guiding the selection of augmented data and the design of augmentation methods.

**Figure 6:**
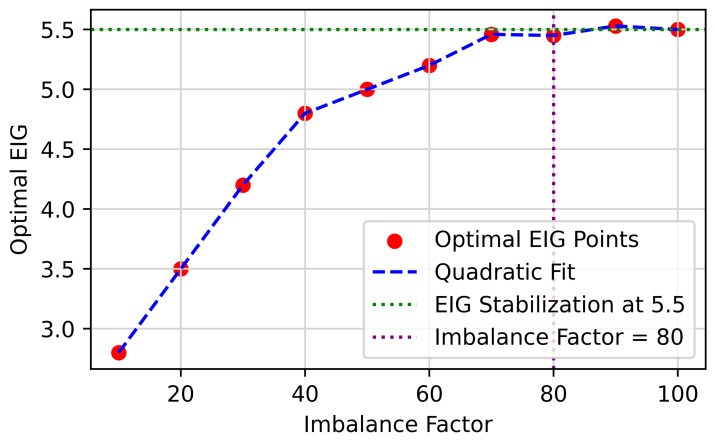
Variation curve of the optimal EIG for augmented data across 10 CIFAR-10-LT datasets with different imbalance factors (IF = i∗10(i=1,2,…,10)).

First, we constructed ten long-tail datasets using CIFAR-10 with imbalance factors of i∗10(i=1,2,…,10). Then, we used CMO to generate 50 groups of augmented samples with different ${\mathrm{EIG}}_{\mathrm{Tail}}$ levels in each long-tail dataset and performed quadratic fitting. We selected the ${\mathrm{EIG}}_{\mathrm{Tail}}$ corresponding to the peak of the quadratic fitting curve in each dataset, resulting in ten points composed of ${\mathrm{EIG}}_{\mathrm{Tail}}$ values and their corresponding imbalance factors. These ten points are plotted in Figure 6, where we observe that as the imbalance factor increases, the growth of the optimal ${\mathrm{EIG}}_{\mathrm{Tail}}$ becomes gradual. When the imbalance factor exceeds 80, the optimal ${\mathrm{EIG}}_{\mathrm{Tail}}$ stabilizes at around 5.5. Therefore, we recommend selecting augmented samples with an ${\mathrm{EIG}}_{\mathrm{Tail}}$ of 5.5 when the imbalance factor of a long-tail dataset is greater than 100.

### 3.7. Selecting Augmented Data with Specified EIGs

Next, we demonstrate the generation of augmented datasets that meet a specified EIG range. To address this, we designed a dynamic subset combination method in Algorithm 2 to approximate the target augmented dataset. This algorithm is based on the following principle: By selecting appropriate subsets from a set of available subsets, we can gradually adjust the EIG value of the augmented dataset to approach the target value. The selection of subsets is based on minimizing the discrepancy between the current EIG value of the augmented dataset and the target EIG value, thereby achieving precise control over the target EIG value. Through this method, we are able to generate effective augmented datasets within the given EIG range, thereby enhancing the performance of models in long-tail datasets. The advantage of this approach is its flexibility to adapt to various data distributions and imbalance factors, ensuring that the augmented samples effectively increase the richness of the category features while avoiding the generation of samples that fall outside the true data distribution.
**Algorithm 2** Subset combination algorithm for target EIG-augmented data**Require:** Data augmentation method (A), initial data $D_{initial}$, target EIG value (${\mathrm{EIG}}_{\mathrm{target}}$), subsets $S=\{S_{1},S_{2},\ldots ,S_{n}\}$, and tolerance ϵ.**Ensure:** Augmented dataset $D_{\mathrm{aug}}$ with EIG close to ${\mathrm{EIG}}_{\mathrm{target}}$1:Initialize $D_{\mathrm{aug}}\leftarrow \emptyset$2:Calculate initial EIG, ${\mathrm{EIG}}_{\mathrm{current}}\leftarrow 0$3:**while** $|{\mathrm{EIG}}_{\mathrm{current}}-{\mathrm{EIG}}_{\mathrm{target}}|>\epsilon$ **do**4:   Find the subset $S_{k}$ from S that minimizes $|\mathrm{EIG}\left(A,D_{initial}\cup D_{\mathrm{aug}}\cup S_{k}\right)-{\mathrm{EIG}}_{\mathrm{target}}|$5:   Update $D_{\mathrm{aug}}\leftarrow D_{\mathrm{aug}}\cup S_{k}$6:   Update ${\mathrm{EIG}}_{\mathrm{current}}\leftarrow \mathrm{EIG}\left(A,D_{initial}\cup D_{\mathrm{aug}}\cup S_{k}\right)$7:   Remove $S_{k}$ from S8:   **if** S is empty **then**9:     **break** Terminate if no more subsets are available10:   **end if**11:**end while**12:**if** $|{\mathrm{EIG}}_{\mathrm{current}}-{\mathrm{EIG}}_{\mathrm{target}}|\leq \epsilon$ **then**13:   **return** $D_{\mathrm{aug}}$14:**else**15:   Report that a suitable combination could not be found16:**end if**

### 3.8. Augmentation Effects on ImageNet-LT

**Figure 7:**
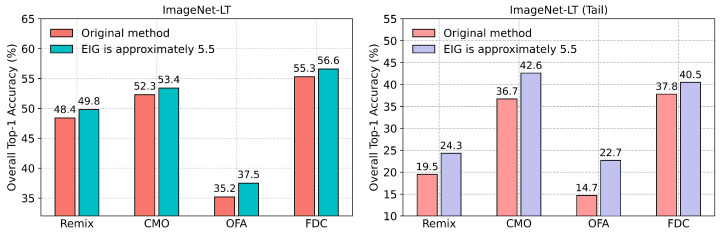
(**Left**): Using the EIG to select augmented samples generated by Remix, CMO, OFA, and FDC in ImageNet-LT significantly improves the overall model performance. (**Right**): Performance improvement in tail classes, using EIG-selected augmented data.

Using Algorithm 2, we obtained augmented samples generated by Remix, CMO, OFA, and FDC, with $EIG_{Tail}$ at around 5.5. We then trained ResNext-50 using both the augmented samples and the original samples. The experimental results are depicted in Figure 7, showing that simply selecting augmented samples with appropriate EIG values can significantly enhance the performance of the original methods without any modifications to the methods or models themselves. Notably, our approach improved the overall performance of OFA by 2.3%. Additionally, we report the performances of all the methods in the tail subset of ImageNet-LT (consisting of categories with fewer than 20 images) in Figure 7. It can be observed that our approach substantially improves the performance of the models in the tail subset. Specifically, our method achieves performance gains of **4.8%**, **5.9%**, **8.0%**, and **2.7%** for Remix, CMO, OFA, and FDC in the tail subset, respectively.

Overall, our experiments demonstrate that guiding data augmentation methods to generate more appropriate samples can further enhance the performances of long-tail models. In the field of long-tail learning, data-centric approaches should be emphasized alongside model-centric approaches to jointly advance the development of the field.

## 4. Conclusions

Most real-world applications involve data with long-tail distributions, leading to biased models. To ensure robust model performance across various scenarios, model-centric approaches, such as reweighted loss functions, transfer learning, and ensemble learning, have been widely proposed. However, as the development of foundational models accelerates, the effectiveness of model-centric methods has gradually diminished. Returning to the essence of the problem, directly improving the data is a fundamental solution to the problems of long-tail learning. The core contribution of this work is the introduction of the effective information gain (EIG), a metric that can guide the design and application of data augmentation methods. Although the impacts of the information gain and distribution shift on data augmentation performance are widely recognized, this study is the first to quantify these two factors and explore how a proper balance between them affects model performance. In the future, we will aim to promote both data-centric and model-centric approaches to advance the field of long-tail learning.

## Data Availability

Data is contained within the article.
